# The Metadata Coverage Index (MCI): A standardized metric for quantifying database metadata richness

**DOI:** 10.4056/sigs.2675953

**Published:** 2012-07-20

**Authors:** Konstantinos Liolios, Lynn Schriml, Lynette Hirschman, Ioanna Pagani, Bahador Nosrat, Peter Sterk, Owen White, Philippe Rocca-Serra, Susanna-Assunta Sansone, Chris Taylor, Nikos C. Kyrpides, Dawn Field

**Affiliations:** 1 Microbial Genomics and Metagenomic Super Program, Department of Energy Joint Genome Institute, Walnut Creek, CA, USA; 2 Institute for Genome Sciences, University of Maryland School of Medicine, Baltimore, MD, USA; 3 The MITRE Corporation, MA, USA; 4 Wellcome Trust Sanger Institute, Wellcome Trust Genome Campus, Cambridge, UK; 5 University of Oxford, Oxford e-Research Centre, Oxford, UK; 6 European Molecular Biology Laboratory (EMBL) Outstation, European Bioinformatics Institute (EBI), Wellcome Trust Genome Campus, Cambridge, UK; 7 Centre for Ecology & Hydrology, Wallingford, Oxfordshire, UK

## Abstract

Variability in the extent of the descriptions of data (‘metadata’) held in public repositories forces users to assess the quality of records individually, which rapidly becomes impractical. The scoring of records on the richness of their description provides a simple, objective proxy measure for quality that enables filtering that supports downstream analysis. Pivotally, such descriptions should spur on improvements. Here, we introduce such a measure - the ‘Metadata Coverage Index’ (MCI): the percentage of available fields actually filled in a record or description. MCI scores can be calculated across a database, for individual records or for their component parts (*e.g.*, fields of interest). There are many potential uses for this simple metric: for example; to filter, rank or search for records; to assess the metadata availability of an *ad hoc* collection; to determine the frequency with which fields in a particular record type are filled, especially with respect to standards compliance; to assess the utility of specific tools and resources, and of data capture practice more generally; to prioritize records for further curation; to serve as performance metrics of funded projects; or to quantify the value added by curation. Here we demonstrate the utility of MCI scores using metadata from the Genomes Online Database (GOLD), including records compliant with the ‘Minimum Information about a Genome Sequence’ (MIGS) standard developed by the Genomic Standards Consortium. We discuss challenges and address the further application of MCI scores; to show improvements in annotation quality over time, to inform the work of standards bodies and repository providers on the usability and popularity of their products, and to assess and credit the work of curators. Such an index provides a step towards putting metadata capture practices and in the future, standards compliance, into a quantitative and objective framework.

## Introduction

“If you cannot measure it, you cannot improve it.”

Lord Kelvin

As the size, number and complexity of bioscience data sets in the public domain continue to grow, appropriate contextualizing of information becomes indispensable. Such ‘halos’ of information are referred to as metadata and include information on how data were collected, processed and analyzed, the nature and state of the biological sample used and the research context. Nowhere is this more relevant than in high-throughput studies using new technologies [1], where the rate of production of data sets is becoming almost unmanageable given current public provision. We are now at a critical stage in which we need to quantify the value of such contextual information.

Metadata considered critical to data interpretation are often referred to as ‘minimum information’ (MI) and this concept has been expressed in various ‘MI checklists’ [2] covering a range of data types including transcriptomics, proteomics, metabolomics and genomics. MI checklists specify the contextual information that should be reported to ensure that studies are (in principle) reproducible and can be compared or combined in an appropriately-informed manner in downstream analyses. Because of the increasing number of such specifications, it behooves the data-sharing community to develop methods to quantify the degree of compliance of databases, individual records or *ad hoc* collections, in order to highlight challenging-to-acquire components of specifications or to quantify improvements in metadata reporting or database content (for example, through curation).

Here we introduce the first, simple metric for evaluating the ‘richness’ of the metadata for any given database (or compliance with a given standard) and a straightforward method to calculate it. The ‘Metadata Coverage Index’ (MCI) is the number of fields in a record for which information is provided, as a percentage of the total fields available. An MCI is no *guarantee* of quality, but given that automated assessment of the semantic content of metadata remains challenging, and that even the correct use of controlled vocabulary terms cannot be a *general* solution as things stand, we are prepared to make the assumption that most annotation constitutes an addition of value to the overall data set and that therefore an MCI is a realizable proxy for the hypothetical Metadata *Quality* Index of a dataset.

An MCI score represents arbitrarily complex contextual information as a simple numerical value. MCI scores can be calculated for individual fields or across collections/databases. While it is clear that some types of metadata carry more value than others, we have made no attempt to model distributions of value across database schemata or MI specifications so that generality for this simplest expression of the metric would be preserved. The weighting of fields according to local or consensus value could be the focus of future work to generate derived versions of MCI reflecting those weightings (*i.e.*, depend on extended validation rules).

To illustrate the calculation of this metric and the usefulness of the concept, we use the MCI to profile the Genomes Online Database (GOLD) [3] and evaluate attempted compliance (*i.e.*, fields filled) with the ‘Minimum Information about a Genome Sequence’ (MIGS) checklist [4] — a part of the MIxS standard [5] from the Genomic Standards Consortium (GSC) [6].

## Materials and Methods

### Data sets

Spreadsheets containing information for genomes from the *Genomic Encyclopedia of Bacteria and Archaea* (GEBA, [7]) and the Human Microbiome Project (HMP) [8] studies, as well as all the genome projects available from GOLD [3] were obtained from the GOLD database.

### Calculation of MCI scores with the MCI Calculator

**Figure 1:**
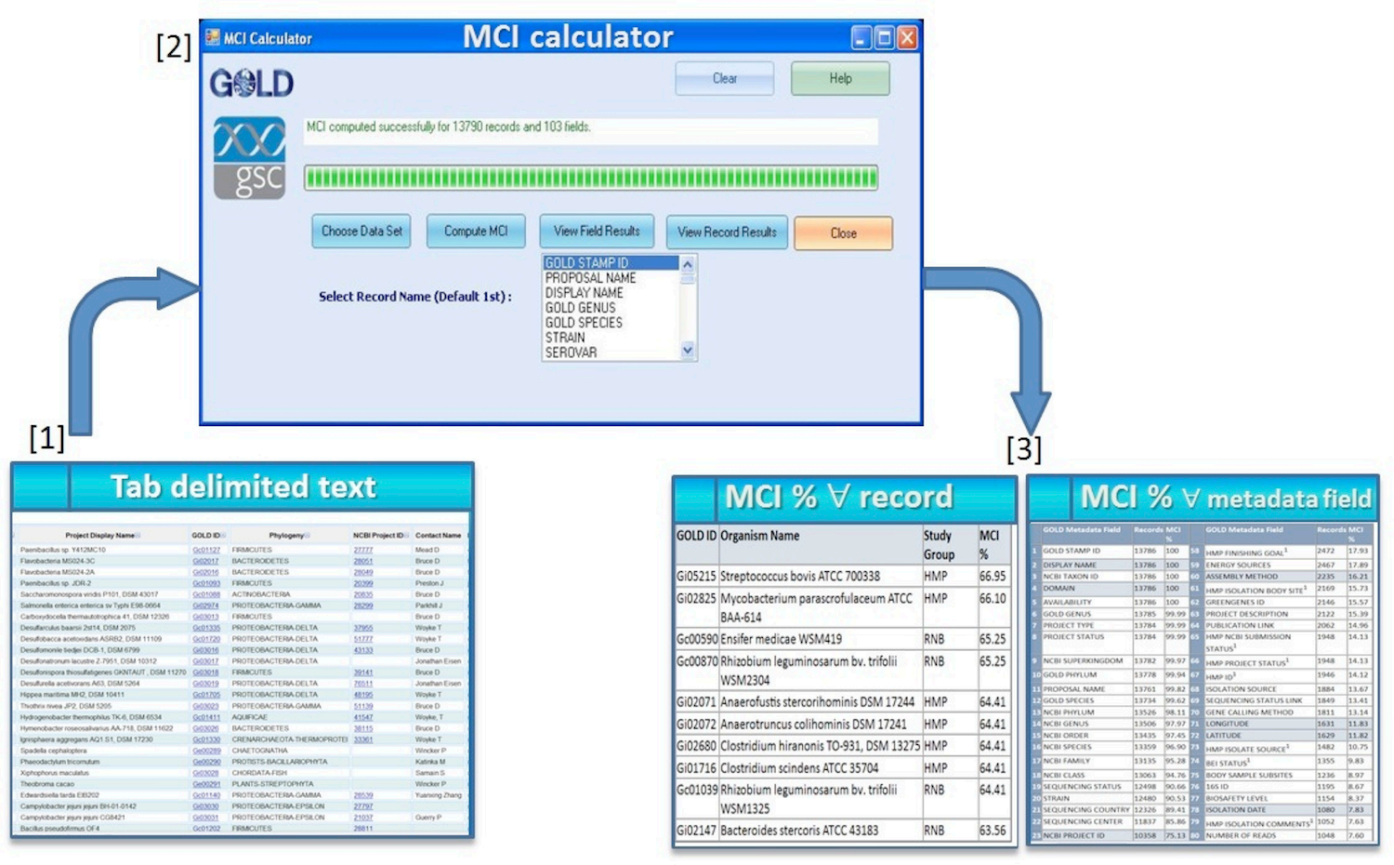
Schematic representation of the MCI calculation procedure.

MCI scores were calculated for each of the above collections as the total number of filled fields expressed as a percentage of the total fields available across all records. Scores were also calculated for individual records and for each field (*i.e*., each variable or column header in a spreadsheet). Note that MCI scores are expressed as percentages, and are therefore size-independent. While the scores could have been calculated using a spreadsheet, the MCI Calculator tool was built to automate the process (Figure 1). As input, it takes any spreadsheet in tabular format. As output, MCI scores are calculated for the whole collection and new spreadsheets are generated containing per-record and per-field scores. The MCI Calculator can be downloaded from the Genomes On Line Database MCI Calculator [9].

### For users: addition of MCI scores to the GOLD database

MCI scores were calculated for all records in GOLD, added to the GOLDCARD pages and offered for use through the GOLD search interface. Thus, MCI scores can now be used to search and sort GOLD records; for example, to retrieve only those records scoring above a certain MCI threshold.

## Results

### Calculating MCI scores and comparison of metadata fields

The GOLD database contains more than two hundred metadata fields across more than thirteen thousand records; well over 2.6 million data points [3]. For the purpose of this study, 113 metadata fields were selected – those applicable to most types of projects – and MCI scores were calculated for them across all genome records in the database (Table 1).

**Table 1:** The list of all selected metadata fields in GOLD (columns 2 and 6)^1^

	**GOLD Metadata Field**	Records	**MCI %**		**GOLD Metadata Field**	Records	**MCI %**
**1**	GOLD STAMP ID	13,786	100	**58**	HMP FINISHING GOAL^2^	2,472	17.93
**2**	DISPLAY NAME	13,786	100	**59**	ENERGY SOURCES	2,467	17.89
**3**	NCBI TAXON ID	13,786	100	**60**	ASSEMBLY METHOD	2,235	16.21
**4**	DOMAIN	13,786	100	**61**	HMP ISOLATION BODY SITE^2^	2,169	15.73
**5**	AVAILABILITY	13,786	100	**62**	GREENGENES ID	2,146	15.57
**6**	GOLD GENUS	13,785	99.99	**63**	PROJECT DESCRIPTION	2,122	15.39
**7**	PROJECT TYPE	13,784	99.99	**64**	PUBLICATION LINK	2,062	14.96
**8**	PROJECT STATUS	13,784	99.99	**65**	HMP NCBI SUBMISSION STATUS^2^	1,948	14.13
**9**	NCBI SUPERKINGDOM	13,782	99.97	**66**	HMP PROJECT STATUS^2^	1,948	14.13
**10**	GOLD PHYLUM	13,778	99.94	**67**	HMP ID^2^	1,946	14.12
**11**	PROPOSAL NAME	13,761	99.82	**68**	ISOLATION SOURCE	1,884	13.67
**12**	GOLD SPECIES	13,734	99.62	**69**	SEQUENCING STATUS LINK	1,849	13.41
**13**	NCBI PHYLUM	13,526	98.11	**70**	GENE CALLING METHOD	1,811	13.14
**14**	NCBI GENUS	13,506	97.97	**71**	LONGITUDE	1,631	11.83
**15**	NCBI ORDER	13,435	97.45	**72**	LATITUDE	1,629	11.82
**16**	NCBI SPECIES	13,359	96.90	**73**	HMP ISOLATE SOURCE^2^	1,482	10.75
**17**	NCBI FAMILY	13,135	95.28	**74**	BEI STATUS^2^	1,355	9.83
**18**	NCBI CLASS	13,063	94.76	**75**	BODY SAMPLE SUBSITES	1,236	8.97
**19**	SEQUENCING STATUS	12,498	90.66	**76**	16S ID	1,195	8.67
**20**	STRAIN	12,480	90.53	**77**	BIOSAFETY LEVEL	1,154	8.37
**21**	SEQUENCING COUNTRY	12,326	89.41	**78**	ISOLATION DATE	1,080	7.83
**22**	SEQUENCING CENTER	11,837	85.86	**79**	HMP ISOLATION COMMENTS^2^	1,052	7.63
**23**	NCBI PROJECT ID	10,358	75.13	**80**	NUMBER OF READS	1,048	7.60
**24**	UPDATE DATE	10,247	74.33	**81**	ORGANISM COMMENTS	948	6.88
**25**	RELEVANCE	9,993	72.49	**82**	METABOLISM	947	6.87
**26**	CONTACT NAME	8,413	61.03	**83**	ISOLATION COMMENTS	874	6.34
**27**	HABITATS	7,979	57.88	**84**	LIBRARY METHOD	778	5.64
**28**	TEMPERATURE RANGE	7,673	55.66	**85**	SEROVAR	774	5.61
**29**	GRAM STAIN	7,341	53.25	**86**	BODY PRODUCTS	723	5.24
**30**	BIOTIC RELATIONSHIP	7,147	51.84	**87**	HOST HEALTH	712	5.16
**31**	CONTACT EMAIL	7,037	51.04	**88**	STRAIN INFO ID	691	5.01
**32**	OXYGEN REQUIREMENT	7,028	50.98	**89**	HMP ISOLATION COMMENTS^2^	690	5.01
**33**	CELL SHAPE	6,748	48.95	**90**	HMP ISOLATION BODY SUBSITE^2^	681	4.94
**34**	DISEASES	6,661	48.32	**91**	SYMBIOTIC RELATIONSHIP	493	3.58
**35**	MOTILITY	6,275	45.52	**92**	SHORT READ ARCHIVE ID	475	3.45
**36**	HOST NAME	5,807	42.12	**93**	INFORMATION URL	465	3.37
**37**	SEQUENCING METHODS	5,636	40.88	**94**	PH	441	3.20
**38**	ISOLATION SITE	5,388	39.08	**95**	IMAGE URL	415	3.01
**39**	SPORULATION	5,187	37.63	**96**	VECTOR	380	2.76
**40**	HOST TAXON ID	5,131	37.22	**97**	SYMBIONT	348	2.52
**41**	GENOME SIZE	4,706	34.14	**98**	SYMBIOTIC INTERACTION	344	2.50
**42**	COMPLETION DATE	4,585	33.26	**99**	ISOLATION PUBMED ID	339	2.46
**43**	CULTURE COLLECTION	4,212	30.55	**100**	HOST GENDER	323	2.34
**44**	CELL ARRANGEMENTS	4,126	29.93	**101**	DEPTH	308	2.23
**45**	PHENOTYPES	4,045	29.34	**102**	SALINITY	281	2.04
**46**	GC PERC	3,693	26.79	**103**	HOST AGE	250	1.81
**47**	GENE COUNT	3,556	25.79	**104**	ISOLATION METHOD	238	1.73
**48**	IN IMG DATABASE	3,453	25.05	**105**	CELL DIAMETER	233	1.69
**49**	PUBLICATION JOURNAL	3,395	24.63	**106**	CELL LENGTH	189	1.37
**50**	SEQUENCING QUALITY	3,286	23.84	**107**	COLOR	157	1.14
**51**	GEO LOCATION	3,265	23.68	**108**	ALTITUDE	94	0.68
**52**	TYPE STRAIN	3,248	23.56	**109**	HOST RACE	72	0.52
**53**	COVERAGE	3,246	23.55	**110**	HOST COMMENTS	50	0.36
**54**	BODY SAMPLE SITES	3,225	23.39	**111**	PROJECT COMMENTS	38	0.28
**55**	ISOLATION COUNTRY	3,140	22.78	**112**	SYMBIONT TAXON ID	36	0.26
**56**	TEMPERATURE OPTIMUM	2,712	19.67	**113**	NCBI ARCHIVE ID	10	0.07
**57**	CONTIG COUNT	2,472	17.93				

^1^with the number of records for each of them (columns 3 and 7), and the MCI % (columns 4 and 8), ordered by the field with highest MCI. Rows in gray belong to the MIGS minimum information checklist that extends what is captured by the INSDC [4] (i.e. full taxonomy is not captured since a reference to a valid NCBI taxid is expected).

^2^ fields relevant only to projects that are part of the HMP study

There are five fields with an MCI score of 100 (fields 1-5 in Table 1). These are the fields filled for all the genome projects in GOLD: essential fields for project registration in the GOLD database. There are seven more fields that have an MCI score greater than 99 (fields 6-13): again, essential fields for project registration – most likely the data are missing due to an error and should be flagged for attention. Some of the fields listed appear to be redundant (e.g. field 6 against 14, or 10 against 13), but when the number of records associated with them is displayed, they make better sense. For example, GOLD has implemented a field named ‘GOLD Genus’ (field 6), in addition to the genus information provided from the NCBI Taxonomy (field 14). This is because genus information is more readily available at the time of project registration with GOLD than it usually through the NCBI taxonomy; also true for phyla. The MCI score for the field ‘NCBI BioProject ID’ is 75%, which implies that 25% of the projects in GOLD are not registered yet with the NCBI BioProject collection. Forty-two percent of projects have ‘Host Name’ information, reflecting the size of the genome projects associated with a specific host organism. 74% of the projects in GOLD have an ‘update’ date (field 24 on Table 1), suggesting that the majority of the projects have been revisited for curation at least once after they were created in the database.

Overall, approximately two thirds of the 113 selected GOLD fields have an MCI score below 50 (fields 33-113). The MCI score across all 113 fields is 34.6. Ten of those fields apply only to projects that are part of the HMP study, and were excluded from subsequent comparisons across different datasets. Twelve fields are part of the MIGS fields as recommended by the GSC [4] (highlighted fields on Table 1). The position of the MIGS fields in the overall list of the 113 fields from GOLD makes clear that these are not the most frequently filled metadata fields across all projects. Only two of the MIGS fields are among the top ten GOLD fields and only six make the top fifty. While the MIGS fields were never likely to be the most populated fields (for example, data for ‘Isolation site’ and ‘Latitude/Longitude’ are frequently not available, even though they are among the most important metadata fields), nonetheless their overall position in the list suggests that a revision may be necessary.

### MCI score comparison of data sets

One advantage of calculating MCI scores as a percentage is that they are size-independent and therefore allow direct comparison across collections. An MCI score captures the proportion of total *possible fields* that are *filled in* (have values) but do not enable a value judgment on the absolute number of values *present* in a particular collection. For comparison, Table 2 shows the MCI scores, along with the total number of records and fields, the maximum number of fields for each collection and the total number of filled values per collection.

**Table 2:** Comparison of MCI scores from the GOLD database. ^1^

	**Project List**	**Field group**	**Fields per Record**	Records	Total Fields	Filled Fields	**MCI %**
**A.**	**1. GEBA**	CORE	103	256	26,368	14,287	54.18
	**2. Complete**			2,040	211,253	109,532	52.00
	**3. HMP**			2,096	215,888	87,007	39.91
	**4. All Projects**			13,790	1,420,370	522,850	37.00
**B.**	**1. Archaea**	CORE	103	340	35,020	16,767	48.00
	**2. Bacteria**			11,233	1,156,999	443,474	38.00
	**3. Eukarya**			2,217	228,351	62,609	27.00
**C.**	**1. GEBA**	MIGS	12	256	3,072	2,102	68.43
	**2. Complete**			2,040	24,612	14,667	59.59
	**3. HMP**			2,096	25,152	9,642	38.34
	**4. All Projects**			13,790	165,480	62,564	37.81
**D.**	**1. HMP**	HMP	10	2,096	20,960	14,673	70.00
**E.**	**1. All-2008**	2008	33	2,905	95,865	59,097	61.65
	**2. All-2010**			5,843	192,819	119,881	62.17
	**3. All-2012**			13,790	455,070	273,805	60.17

^1^ Note that if all variables in a database or collection apply to all records, then ‘total fields’ is equal to records multiplied by fields. If some variables are specific to a subset of records then the total number of possible fields will be smaller.

We have created nine distinct project collections from GOLD (Project list column on Table 2) and organized them in three separate groups, enabling comparison of the richness of various slices of the full database. Each comparison is meaningful only within its own group. For example, the ‘GEBA’ collection comprises 256 genome projects, all part of the GEBA study. The collection ‘Complete’ refers to the 2,040 complete genome projects available in GOLD; ‘HMP’ refers to the 2,096 projects selected for sequencing under the HMP study. The collection ‘All projects’ encompasses the currently available 13,790 isolate genome projects in GOLD, while ‘Archaea’, ‘Bacteria’ and ‘Eukarya’ relate to the corresponding phylogenetic subgroups. Each project collection group is characterized by the specific number and type of fields selected for the comparison. This is essential in order to select fields that would be applicable for all the projects within a list. Accordingly, all the HMP related fields were excluded from the total number of fields used in this study, thus creating a set of 103 fields that apply to all project lists (CORE group). In a similar manner, the ten HMP-specific fields have been grouped to compose the HMP group, while the 12 MIGS fields comprise the MIGS group of fields (all shown on the column Field group on Table 2).

Comparing the GEBA collection against the complete genomes, the HMP and the all-projects lists, using the core 103 metadata fields (group A on Table 2), reveals that GEBA has the best-curated project metadata, having the highest MCI score (54.18%). This reflects the emphasis given to the collection and curation of metadata for this project, suggesting a formal role for MCI as a performance metric. The availability of SIGS compliant genome reports for all the completed GEBA genomes, certainly had a pivotal role in providing a well curated and standardized source of key metadata for those projects [10]. In terms of metadata coverage across different phylogenetic groups within the GOLD dataset (group 2, on Table 2), archaeal and bacterial subsets of the data had higher MCI scores than eukaryotes, reflecting the value of more-detailed curation of the microbial genome projects for GOLD. Likewise, subsets of data compliant with the MIGS standard fields also had relatively higher general MCI scores, with the GEBA list reaching 68% of metadata coverage (group C on Table 2), almost 10% more than the average complete genome. Finally, within the HMP project list the HMP fields have a high 70% MCI score (group D on Table 2).

## Improvements in MCI scores over time

MCI scores can be used to compare collections and to quantify incremental increases in the richness of metadata over time. To illustrate this we compared the information contained in the GOLD database in 2008 [11], 2010 [12] and in 2012. The 2008 publication of GOLD reported a list of 45 metadata fields and the number of projects associated with those fields [11], while the 2010 publication of GOLD reported 105 variables and the number of projects for which information was available [12]. We selected a common set of 33 fields across the three sets and calculated the MCI scores for those (group E on Table 2). The results of this comparison revealed that the overall MCI score has remained stable around 60%, although the total number of records has been doubling every two years. This raises the question of whether more recent submitters have tended to report more metadata, which would be indicative of increased acceptance of the value of appropriate metadata. However, since the majority of the data available from the GOLD database are not provided from the submitters but rather collected and curated in the database, it is hard to accurately address that question with these data.

## Calculating MCI Scores for Records and Fields

**Figure 2:**
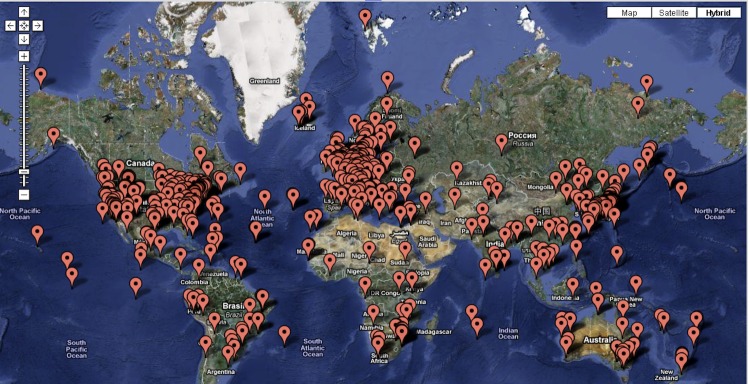
MCI scores are implemented in the GOLD database. MCI scores can be seen on the GOLDCARDS for each entry and are including in the advanced search option. For example, all entries with an MCI score > 50 are shown on the map below.

MCI scores can be calculated for individual records or fields (variables) in a given dataset. This allows variation in MCI scores to be used to compare, sort and search records within datasets, or to select subsets. To show the utility of calculating MCI scores per record, MCI scores were included in the GOLD database. Using the advanced search option, users can now select records based on MCI score. For example, Figure 2 shows all entries with MCI scores > 50 on a world map, using associated metadata on the country of origin. The first ten projects in GOLD ranked by MCI score are shown in Table 3. Interestingly, six are part of the HMP study, while the remaining four projects are part of the Root Nodulating Bacteria (RNB) study running at the DOE Joint Genome Institute [13]. These findings reveal that although the entire list of 2,096 HMP projects has a relatively low MCI score (39.91%), some of the best-curated projects belong to this group. This is expected, given that the MCI score of an entire dataset is the average score of all the records comprising that dataset. If some of the records are poorly curated, then the overall MCI score of that dataset will be lower. The HMP dataset, which is comprised of 2,096 records, is an excellent set to demonstrate this issue. This group may have some of the best curated records, as shown on Table 3, but, it includes a large number of records (about 20% of the total) that represent targeted projects, for which very limited metadata is available.

**Table 3:** The list of the genome projects in GOLD with the top 10 MCI scores

**GOLD ID**	**Organism Name**	**Study Group**	**MCI %**
Gi05215	*Streptococcus bovis* ATCC 700338	HMP	66.95
Gi02825	*Mycobacterium parascrofulaceum* ATCC BAA-614	HMP	66.10
Gc00590	*Ensifer medicae* WSM419	RNB	65.25
Gc00870	*Rhizobium leguminosarum* bv. trifolii WSM2304	RNB	65.25
Gi02071	*Anaerofustis stercorihominis* DSM 17244	HMP	64.41
Gi02072	*Anaerotruncus colihominis* DSM 17241	HMP	64.41
Gi02680	*Clostridium hiranonis* TO-931, DSM 13275	HMP	64.41
Gi01716	*Clostridium scindens* ATCC 35704	HMP	64.41
Gc01039	*Rhizobium leguminosarum* bv. *trifolii* WSM1325	RNB	64.41
Gi02147	*Bacteroides stercoris* ATCC 43183	RNB	63.56

**Figure 3:**
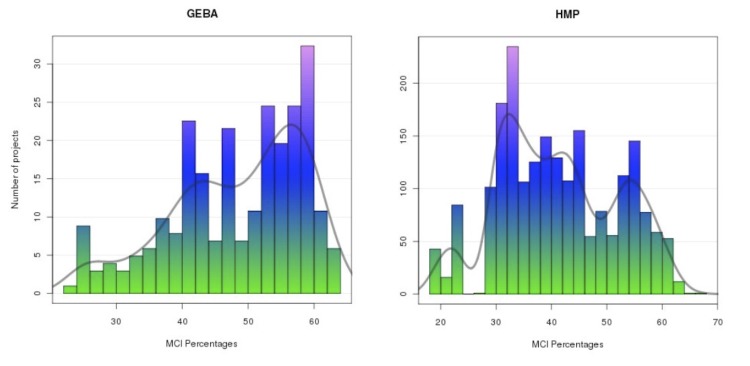
Distribution of the MCI percentages for the GEBA and HMP groups.

Accordingly, the above discussion points out that an MCI score is useful when applied to large datasets: it can provide the average score across all the records as well as the distribution of the scores across the records. To demonstrate this, we plot the distribution of the MCI scores across the HMP and GEBA datasets, for each of their corresponding records. As shown on Figure 3, this distribution reveals that the HMP dataset has indeed a larger number of records that currently are characterized with lower MCI score, compared to the GEBA dataset.

## Discussion

We have described a new metric characterizing the richness of metadata in a given database, record or other collection. High MCI scores identify the most commonly-filled fields in existing records and could be used to automatically select the most useful fields for display in tables or web interfaces (*i.e.*, the richest or most commonly-complete subsets of the data), or to empirically validate the content of a ‘minimum information’ specification [2]. The fields most frequently filled in a given collection are good candidates to be formalized by a community as a ‘core’ requirement. If there is a mismatch – for example, if fields marked as ‘core’ in a standard are difficult to collect, or those with 100% compliance are not included – it suggests that standard might need to be revised; for example, with respect to the GSC definition of new habitat-specific metadata fields (‘environmental packages’) [5].

MCI scores, as defined here, only take into account simple presence or absence of values. It is clearly important to make sure these values are valid (for example not uninformative ‘placeholders’ entered into required fields by reluctant data submitters or otherwise inappropriate information). Likewise, sheer quantity of metadata is not always necessarily optimal and care needs to be taken in both generating and interpreting MCI scores in a manner that is appropriate to the interpretation of the data at hand. MCI scores are best used when the exact variables in the total list of expected fields are well defined and transparent to the user (i.e. ideally selected from a minimum standard).

MCI scores will ideally be used to make targeted improvements to databases over time. They could also be used over time to track the evolution of databases and their contents, for example, to signal significant updates in content even when the total number of entries remains the same, to report progress to funders, or to reward the work of curators who contribute the relevant information. Methods that aid in defining the pivotal contributions of curators and rewarding their efforts to the wider community are needed.

MCI scores could be further refined in several ways; for example, to include only fields matching certain criteria (*e.g.*, string, number, regular expression-compliant, or curated *versus* calculated values), or those using terms from recognized ontologies. This would be particularly useful for judging compliance with a given standard like MIGS – since free text is not allowed, formal validation could be done using, for example, GCDML [14] (for genomics) or the ISA-Tab (multi-omic) format [15]. MCI scores could also be broken down to cover ‘required’ and ‘optional’ fields separately.

Further refinement of MCI scores would require more thorough validation of metadata, making maximum use of mappings between minimal information requirements, recommended terminologies and any formats used. New efforts emerging from the community are laying the basis for such a multi-dimensional validation process: Data standardization efforts such as the ISA Commons [16] offer common metadata tracking frameworks that can better underpin and facilitate the development of improved validation methods.

Where databases such as PRIDE [17] allow free use of controlled vocabularies to extend records (*i.e.*, *user*-defined fields), the list of identifiable fields may appear disproportionately large (each term used becomes a field, making for a *very* sparse matrix). MCI requires adaptation for use in such data structures, but even in basic form can be useful in defining whether one or more core (minimum) sets of metadata can be identified (subsets of the data with MCI scores well above average).

When calculating MCI scores, it is important to consider that databases may also contain markedly different subsets (for example, delineated by technique or taxon); appropriate partitioning of records before calculation would address this.

In summary, the MCI scores individual records according to the completeness of their metadata and of their component fields, providing valuable insights into the provenance, value and cost of those records. As such, it serves as an objective and quantifiable metric for metadata capture and highlights the scholarly work required to develop curated collections [18]. We look forward to the time when other databases utilize MCI scores, as it will also serve to provide a qualitative assessment between these resources.

## References

[1] Field D Sansone SA Collis A Booth T Dukes P Gregurick SK Kennedy K Kolar P Kolker E Maxon M 'Omics Data Sharing. Science 2009; 326:234-236 10.1126/science.1180598 19815759 PMC2770171

[2] Taylor CF Field D Sansone SA Aerts J Apweiler R Ashburner M Ball CA Binz PA Bogue M Booth T Promoting coherent minimum reporting guidelines for biological and biomedical investigations: the MIBBI project. Nat Biotechnol 2008; 26:889-896 10.1038/nbt.1411 18688244 PMC2771753

[3] Pagani I Liolios K Jansson J Chen IM Smirnova T Nosrat B Markowitz VM Kyrpides NC The Genomes On Line Database (GOLD) v.4: status of genomic and metagenomic projects and their associated metadata. Nucleic Acids Res 2012; 40:D571-D579 10.1093/nar/gkr1100 22135293 PMC3245063

[4] Field D Garrity G Gray T Morrison N Selengut J Sterk P Tatusova T Thomson N Allen MJ Angiuoli SV The minimum information about a genome sequence (MIGS) specification. Nat Biotechnol 2008; 26:541-547 10.1038/nbt1360 18464787 PMC2409278

[5] Yilmaz P Kottmann R Field D Knight R Cole JR Amaral-Zettler L Gilbert JA Karsch-Mizrachi I Johnston A Cochrane G Minimum information about a marker gene sequence (MIMARKS) and minimum information about any (x) sequence (MIxS) specifications. Nat Biotechnol 2011; 29:415-420 10.1038/nbt.1823 21552244 PMC3367316

[6] Field D Amaral-Zettler L Cochrane G Cole JR Dawyndt P Garrity GM Gilbert J Glöckner FO Hirschman L Karsch-Mizrachi I The Genomic Standards Consortium (GSC). PLoS Biol 2011; 9:e1001088 10.1371/journal.pbio.1001088 21713030 PMC3119656

[7] Wu D Hugenholtz P Mavromatis K Pukall R Dalin E Ivanova NN Kunin V Goodwin L Wu M Tindall BJ A phylogeny-driven genomic encyclopaedia of Bacteria and Archaea. Nature 2009; 462:1056-1060 10.1038/nature08656 20033048 PMC3073058

[8] Peterson J Garges S Giovanni M McInnes P Wang L Schloss JA Bonazzi V McEwen JE Wetterstrand KA Deal C The NIH Human Microbiome Project. Genome Res 2009; 19:2317-2323 10.1101/gr.096651.109 19819907 PMC2792171

[9] Genomes On Line Database MCI. Calculator. http://genomesonline.org/SetupMCICalculator.msi

[10] Garrity GM Field D Kyrpides NC Standards in Genomic Sciences. Stand Genomic Sci 2009; 1:1-2 10.4056/sigs.34251 21475581 PMC3072088

[11] Liolios K Chen IM Mavromatis K Tavernarakis N Hugenholtz P Markowitz V Kyrpides NC The Genomes On Line Database (GOLD) in 2009: status of genomic and metagenomic projects and their associated metadata. Nucleic Acids Res •••; 38:D346-D354 10.1093/nar/gkp848 19914934 PMC2808860

[12] Liolios K Mavromatis K Tavernarakis N Kyrpides NC The Genomes On Line Database (GOLD) in 2007: status of genomic and metagenomic projects and their associated metadata. Nucleic Acids Res 2008; 36:D475-D479 10.1093/nar/gkm884 17981842 PMC2238992

[13] http://genome.jgi-psf.org/programs/bacteria-archaea/GEBA-RNB.jsf.

[14] Kottmann R Gray T Murphy S Kagan L Kravitz S Lombardof T Field D Glockner FO Genomic Standards Consortium A standard MIGS/MIMS compliant XML Schema: toward the development of the Genomic Contextual Data Markup Language (GCDML). Omics: a journal of integrative biology 2008; 12:115-121 10.1089/omi.2008.0A10 18479204

[15] Rocca-Serra P Brandizi M Maguire E Sklyar N Taylor C Begley K Field D Harris S Hide W Hofmann O ISA software suite: supporting standards-compliant experimental annotation and enabling curation at the community level. Bioinformatics 2010; 26:2354-2356 10.1093/bioinformatics/btq415 20679334 PMC2935443

[16] Sansone SA Rocca-Serra P Field D Maguire E Taylor C Hofmann O Fang H Neumann S Tong W Amaral-Zettler L Toward interoperable bioscience data. Nat Genet 2012; 44:121-126 10.1038/ng.1054 22281772 PMC3428019

[17] Jones P Côté RG Cho SY Klie S Martens L Quinn AF Thorneycroft D Hermjakob H PRIDE: new developments and new datasets. Nucleic Acids Res 2008; 36:D878-D883 10.1093/nar/gkm1021 18033805 PMC2238846

[18] Howe D Costanzo M Fey P Gojobori T Hannick L Hide W Hill DP Kania R Schaeffer M St Pierre S Big data: The future of biocuration. Nature 2008; 455:47-50 10.1038/455047a 18769432 PMC2819144

